# Supplementary material for: Modeling glioblastoma heterogeneity as a dynamic network of cell states
Source: Mol Syst Biol. 2021 Sep 16;17(9):e10105. doi: 10.15252/msb.202010105 (PMC8444284; doi:10.15252/msb.202010105)
Supplement: Supplementary file 6 — Source Data for Figure 5 [file MSB-17-e10105-s004.zip › Figure5A_sourcedata/GSEA_3071/nosigdb_stateVI.GseaPreranked.1622795169056/gsea_report_for_na_pos_1622795169056.html]

Report for na\_pos 1622795169056 [GSEA]

| GS  follow link to MSigDB | GS DETAILS | SIZE | ES | NES | NOM p-val | FDR q-val | FWER p-val | RANK AT MAX | LEADING EDGE || 1 | LARSSON\_STATE\_1 | Details ... | 62 | 0.46 | 3.04 | 0.000 | 0.000 | 0.000 | 383 | tags=48%, list=11%, signal=53% |
| 2 | RICHARDS\_VERHAAK\_CANCERCELL\_2010\_MESENCHYMAL | Details ... | 71 | 0.42 | 2.83 | 0.000 | 0.000 | 0.000 | 569 | tags=48%, list=16%, signal=56% |
| 3 | SUVA\_MES2\_PMID\_31327527 | Details ... | 22 | 0.47 | 2.23 | 0.000 | 0.004 | 0.010 | 1185 | tags=82%, list=33%, signal=121% |
| 4 | RICHARDS\_NEFTEL\_CELL\_2019\_MES2 | Details ... | 22 | 0.47 | 2.22 | 0.000 | 0.003 | 0.010 | 1185 | tags=82%, list=33%, signal=121% |
| 5 | IVY\_CTPAN | Details ... | 68 | 0.33 | 2.21 | 0.000 | 0.003 | 0.012 | 416 | tags=41%, list=11%, signal=46% |
| 6 | FELDMAN\_2020\_LATEG1 | Details ... | 61 | 0.34 | 2.20 | 0.000 | 0.002 | 0.012 | 437 | tags=41%, list=12%, signal=46% |
| 7 | DALMO\_EXTINCTION\_UNPUB | Details ... | 19 | 0.49 | 2.16 | 0.006 | 0.003 | 0.015 | 477 | tags=58%, list=13%, signal=66% |
| 8 | SINGH\_SOX2\_DOWNREGULATED\_TRANSCRIPTS\_PMID\_28122245 | Details ... | 59 | 0.32 | 2.10 | 0.000 | 0.004 | 0.024 | 412 | tags=32%, list=11%, signal=36% |
| 9 | WANG\_MESENCHYMAL\_PMID\_28697342 | Details ... | 19 | 0.49 | 2.09 | 0.007 | 0.003 | 0.024 | 477 | tags=58%, list=13%, signal=66% |
| 10 | SEGERMAN\_MULTITHERAPY\_RESISTANCE\_FIG5A\_PMID\_27974212 | Details ... | 17 | 0.47 | 1.97 | 0.004 | 0.007 | 0.058 | 913 | tags=65%, list=25%, signal=86% |
| 11 | RICHARDS\_LIDDELOW\_NATURE\_2017\_A2\_REACTIVE\_ASTROCYTES | Details ... | 19 | 0.43 | 1.93 | 0.007 | 0.009 | 0.077 | 404 | tags=58%, list=11%, signal=65% |
| 12 | NB\_KI\_CLUSTER12 | Details ... | 27 | 0.39 | 1.92 | 0.009 | 0.008 | 0.079 | 452 | tags=41%, list=12%, signal=46% |
| 13 | NB\_KI\_CLUSTER11 | Details ... | 32 | 0.34 | 1.78 | 0.017 | 0.019 | 0.176 | 347 | tags=31%, list=10%, signal=34% |
| 14 | SUVA\_MES1\_PMID\_31327527 | Details ... | 26 | 0.34 | 1.67 | 0.016 | 0.034 | 0.327 | 471 | tags=46%, list=13%, signal=53% |
| 15 | RICHARDS\_NEFTEL\_CELL\_2019\_MES1 | Details ... | 26 | 0.34 | 1.63 | 0.028 | 0.040 | 0.393 | 471 | tags=46%, list=13%, signal=53% |
| 16 | RICHARDS\_NOWAKOWSKI\_SCIENCE\_2017\_MICROGLIA\_UPREG | Details ... | 309 | 0.18 | 1.59 | 0.000 | 0.049 | 0.473 | 583 | tags=28%, list=16%, signal=31% |
| 17 | NB\_KI\_CLUSTER13 | Details ... | 69 | 0.23 | 1.56 | 0.018 | 0.056 | 0.531 | 854 | tags=42%, list=23%, signal=54% |
| 18 | DOROSZKO\_U3180MG\_CLUSTER\_4 | Details ... | 71 | 0.22 | 1.49 | 0.035 | 0.077 | 0.670 | 974 | tags=48%, list=27%, signal=64% |
| 19 | ROSEN\_KRONA\_TI\_UNPUBL | Details ... | 36 | 0.23 | 1.31 | 0.138 | 0.191 | 0.952 | 718 | tags=36%, list=20%, signal=45% |
| 20 | NB\_KI\_CLUSTER15 | Details ... | 68 | 0.18 | 1.27 | 0.134 | 0.219 | 0.980 | 924 | tags=40%, list=25%, signal=52% |
| 21 | ANASTASSIOU\_MULTICANCER\_INVASIVENESS\_PMID\_22208948 |  | 32 | 0.21 | 1.10 | 0.354 | 0.448 | 1.000 | 485 | tags=28%, list=13%, signal=32% |
| 22 | NB\_KI\_CLUSTER3 |  | 69 | 0.16 | 1.09 | 0.333 | 0.449 | 1.000 | 508 | tags=20%, list=14%, signal=23% |
| 23 | RICHARDS\_NOWAKOWSKI\_SCIENCE\_2017\_GLYC\_UPREG |  | 262 | 0.12 | 1.07 | 0.294 | 0.453 | 1.000 | 838 | tags=31%, list=23%, signal=37% |
| 24 | NB\_KI\_CLUSTER22 |  | 51 | 0.16 | 1.01 | 0.461 | 0.534 | 1.000 | 569 | tags=29%, list=16%, signal=34% |
| 25 | KRONA\_ROSEN\_PERIVASCULAR\_INVASION |  | 34 | 0.19 | 0.98 | 0.490 | 0.565 | 1.000 | 652 | tags=32%, list=18%, signal=39% |
| 26 | RICHARDS\_NEFTEL\_CELL\_2019\_G2\_M |  | 19 | 0.21 | 0.91 | 0.580 | 0.676 | 1.000 | 199 | tags=16%, list=5%, signal=17% |
| 27 | SUVA\_G2M\_PMID\_31327527 |  | 19 | 0.21 | 0.91 | 0.582 | 0.659 | 1.000 | 199 | tags=16%, list=5%, signal=17% |
| 28 | ALMSTEDT\_17Q\_MSIGDB |  | 125 | 0.12 | 0.89 | 0.745 | 0.663 | 1.000 | 816 | tags=30%, list=22%, signal=38% |
| 29 | DOROSZKO\_U3054MG\_CLUSTER\_9 |  | 177 | 0.10 | 0.87 | 0.816 | 0.669 | 1.000 | 717 | tags=25%, list=20%, signal=30% |
Table: Gene sets enriched in phenotype **na **[plain text format]****
